# Surface Microstructure Study on Corona Discharge-Treated Polyethylene Using Positron Annihilation Spectroscopy

**DOI:** 10.3390/molecules29174147

**Published:** 2024-08-31

**Authors:** Jingjing Li, Zhiwei Shen, Liuyang Tie, Tianyuan Long, Qiyue Zhong, Xi Chen, Chongshan Yin, Liguo Liufu, Xianhao Huang, Bangyun Xiong, Xibo Li, Chongxiong Duan, Chunqing He

**Affiliations:** 1 Guangdong Key Laboratory for Hydrogen Energy Technologies, School of Materials Science and Hydrogen Energy, Foshan University, Foshan 528000, China; jjli@fosu.edu.cn (J.L.); shenzw8106@163.com (Z.S.); tieliuyang311@163.com (L.T.); 13631450402@163.com (T.L.); kop0526@outlook.com (Q.Z.); cx18023404833@163.com (X.C.); 18520322773@163.com (L.L.); 13674041967@163.com (X.H.); xiongbangyun@163.com (B.X.); cechxduan@fosu.edu.cn (C.D.); 2 Hunan Provincial Key Laboratory of Flexible Electronic Materials Genome Engineering, School of Physics and Electronic Science, Changsha University of Science and Technology, Changsha 410114, China; c.sh.yin@foxmail.com; 3 Department of Physics, Jinan University, Guangzhou 510632, China; 4 Key Laboratory of Nuclear Solid-State Physics Hubei Province, School of Physics and Technology, Wuhan University, Wuhan 430072, China

**Keywords:** corona discharge, positron annihilation, free volume, *S* parameter, hydrophilicity

## Abstract

The microstructure and chemical properties of the corona discharge process could provide an effective method for predicting the performance of high-voltage cable insulation materials. In this work, the depth profile of the microstructure and chemical characteristics of corona discharge-treated PE were extensively investigated using Doppler broadening of position annihilation spectroscopy accompanied with positron annihilation lifetime spectroscopy, attenuated total reflectance Fourier transform infrared spectra, Raman spectra and contact angle measurement. By increasing corona discharge duration, the oxygen-containing polar groups, including hydroxyl, carbonyl and ester groups, strongly contribute to the deterioration of hydrophobicity and the enhancement of hydrophilicity. And the mean free volume size, with a broadening distribution, decreases slightly. The line shape *S* parameter decreases because of the decrease in free volume elements and the appearance of oxygen-containing groups. Also, the thickness of the degradation layer, determined from the *S* parameter with positron injection depth, increases and diffuses into the PE matrix. A linear *S-W* plot within the degradation layer of different corona treatment duration samples indicates the defect type does not change. The *S* parameter decreases and the *W* parameter increases with an increasing corona duration. Using a slow positron beam, the nondestructive probe can be used to profile the microstructure and chemical environment across the corona discharge damage depth, which is beneficial for investigating the surface and interfacial insulation materials.

## 1. Introduction

Polyethylene (PE), with chemically inert properties and a low dielectric constant, has been widely used for electrical insulation materials in power transmission systems such as low-voltage, high-voltage and ultrahigh-voltage cable. Electrical aging and dielectric local heating are the main reasons for the premature failure of insulators operating at high voltage. Long-term corona discharge treatment leads to the deterioration or even failure of insulating materials. The main theory of corona discharge contains space charge, electron collision theory, the ozone erosion effect and dielectric heating [1,2,3]. The physical and chemical properties of a material surface vary significantly after corona discharge treatment, involving changes in free energy and surface wettability [4], surface charge generation and recombination [5], surface degradation characteristics [6] and the dynamic response of space charge inside the dielectric [7]. A study focusing on the microstructure and chemical properties during the corona discharge process could provide an effective method for predicting the performance of insulating materials. Many other probing techniques, including X-ray, neutron scattering electron microscopy, small-angle neutron scattering (SANS), dynamical mechanical analysis (DMA), X-ray diffraction (XRD) and differential scanning calorimetry (DSC), are used to characterize the structure of polymer materials at different lengths and infer changes in the polymer structure in an indirect way [8]. However, positron annihilation spectroscopy adopts a direct way. It can profile the corona discharge damage depth in corona discharge materials, since the penetration depth of positrons can be controlled via controlling the injected positron energy. Positron annihilation technology is a powerful non-destructive probe tool used to investigate the microstructure and chemical characteristics of different materials containing vacancies, vacancy clusters, free volumes, porous structures, interfaces, surfaces, as well as some other films [9,10,11,12,13]. An energetic positron injected into condensed matter will annihilate with an electron or become positronium (Ps) after slowing down to a thermal state through inelastic collisions with surround atomic cores. Ps, a bound state of a positron and an electron, with a Bohr diameter of 0.106 nm, contains triple-state ortho-Ps (*o*-Ps) with parallel spins and singlet-state para-Ps (*p*-Ps) with antiparallel spins. In vacuum, *o*-Ps annihilates into 3γ rays with an intrinsic lifetime of 142 ns, and *p*-Ps annihilates into 2γ rays with a very short intrinsic lifetime of 125 ps. *o*-Ps is highly sensitive to low-electron-density areas such as vacancies, free volumes and holes, and it is easily trapped therein. It picks up an external electron of antiparallel spin on the wall of the hole and annihilates into 2γ rays, with a lifetime shortened to a few nanoseconds, a process known as pick-off annihilation. Generally, for polymer materials, a positron annihilation lifetime spectrum (PALS) is resolved into three or four components, and it is characterized by lifetimes (τ_1_: ~0.125, τ_2_: 0.3~0.5 and a long lifetime τ_3_ of 1~10 ns) and corresponding intensities (I_1_, I_2_, I_3_). The long-lifetime component is closely related to the hole’s size and relative quantity. A semiempirical Tao–Eldrup equation [14,15] based on a simple quantum mechanical model clearly describes the relation between the *o*-Ps lifetime (*τ*_3_) and the mean radius (*R*) of a hole smaller than 1 nm:

(1) $$\tau_{3}=\frac{1}{2}{\left(1-\frac{R}{R+\Delta R}+\frac{1}{2\pi }\sin\left(\frac{2\pi R}{R+\Delta R}\right)\right)}^{-1},$$

where *R* is the average radius of the free volume and ΔR = 1.66 Å (an empirical parameter).

Doppler broadening spectroscopy (DBS) is based on the detection of 511 keV γ energy spectroscopy when positrons are annihilated with either valance or core electrons, which reflects the momentum distribution of the electrons participating. Annihilating with valance electrons can be described by the line shape parameter *S*, which is defined as the ratio of the annihilation events of the central region to the total area of the 511 keV annihilation peak. The other parameter, named *W*, which is defined as the ratio of the annihilation events of the wing regions to the total peak, represents the characteristics of positron annihilation with surrounding core electrons. Generally speaking, *S* and *W* can be used to detect the variations in sub-nanometer defect sizes and quantities, as well as the chemical characteristics of materials. In most of the polymeric materials, the *S* parameter is sensitive to the variation of free volumes or holes and their concentration. And it represents the relative contribution of the low-momentum part of the positron–electron annihilation radiation [16]. For materials with more vacancy-type defects and free volume holes, the *S* parameter will exhibit a higher value [17,18]. Both the increase in the self-annihilation of *p*-Ps and the information probability of Ps contribute to the narrowing of the 511 keV annihilation peak, and an increase in the *S* parameter will occur [19].

A depth profile study of a polymer sample (surface, films and interface) [17,20,21] was successfully carried out using the slow positron annihilation technique via controlling the injected positron energy after moderating, converging and accelerating. And the mean implantation depth of positrons depending on the injected positron energy in materials is described as follows [22]:

(2) $$Z_{m}=40\times \frac{E^{1.6}}{\rho },$$

where *Z*_m_ is the mean injected depth (nm), *E* is the injected positron energy (keV) and *ρ* is the density of polyethylene in this work (*ρ* of PE is 0.94 g/cm^3^).

The main aim was to investigate the depth profile microstructure and chemical properties of the corona discharge process at an early stage. In this study, the PE samples were corona discharge-treated for varying durations using a “needle-plate” system at 5 kV and 50 Hz. And the microstructure, chemical properties and hydrophilicity were extensively studied using ATR-FTIR, Raman spectroscopy, contact angle, PALS and DBS.

## 2. Results and Discussion

### 2.1. Chemical Properties of Corona Discharge-Treated PE

**Figure 1:**
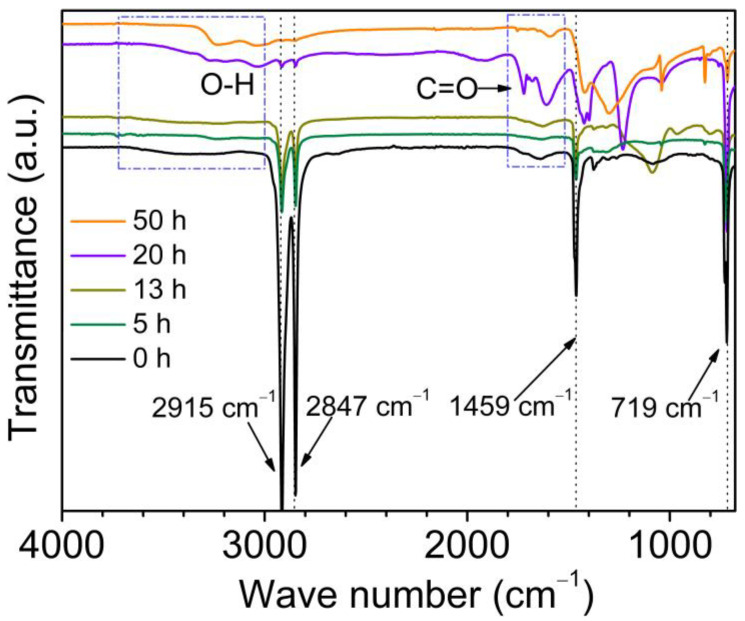
ATR-FTIR spectra of PE before and after different corona discharged duration.

To extensively study the chemical properties of corona discharge-treated PE, ATR-FTIR spectrometry was used. As shown in Figure 1, there are four absorption bands of methylene (-CH_2_-) for untreated PE. Asymmetric and symmetric stretching vibration as well as the bending and rocking vibration of the C-H bond are observed at 2915 cm^−1^, 2847 cm^−1^, 1459 cm^−1^ and 719 cm^−1^, respectively. These findings are consistent with those reported in the literature [23]. After corona discharge treatment, the absorption band intensity decreases significantly with increasing corona discharge duration. Further, the absorption band at 3700~3000 cm^−1^ is related to the hydroxyl (O-H) stretching vibration. It suggests that the corona discharge treatment contributes to the formation of hydroxyl radicals. The stretching vibration of carbonyl (C=O) appears at 1720 cm^−1^ and 1592 cm^−1^ [24]. And the vibration of ester group (-O-C=O-) appears at 1247 cm^−1^ and 1039 cm^−1^. With increasing corona discharge treatment duration from 20 h to 50 h, a much weaker carbonyl group is noticed. Another interesting phenomenon is the vibration band of the ester group showing noticeable differences between the 20 h- and 50 h-treated sample. An absorption band can also be observed in the region of 1300–1421 cm^–1^, indicative of the formation of a C-N bond. Corona discharge damage on the hydrocarbon backbone of PE may produce carbon radicals, which react with nitrogen in air. The results suggest the chemical modification generated by corona discharge treatment, as evidenced by the introduction of oxygen-containing groups or even a C-N bond within the carbon-hydrogen backbone.

Considering the methylene contributes to poor wettability, these new generated oxygen-containing polar groups suggest that the hydrocarbons chain structure of PE is destroyed by corona discharge treatment and the surface is chemically modified by corona discharge for the formation of carbon radicals, unstable hydroperoxides and the oxygen-containing polar groups [25]. And the wettability will be improved. 

**Figure 2:**
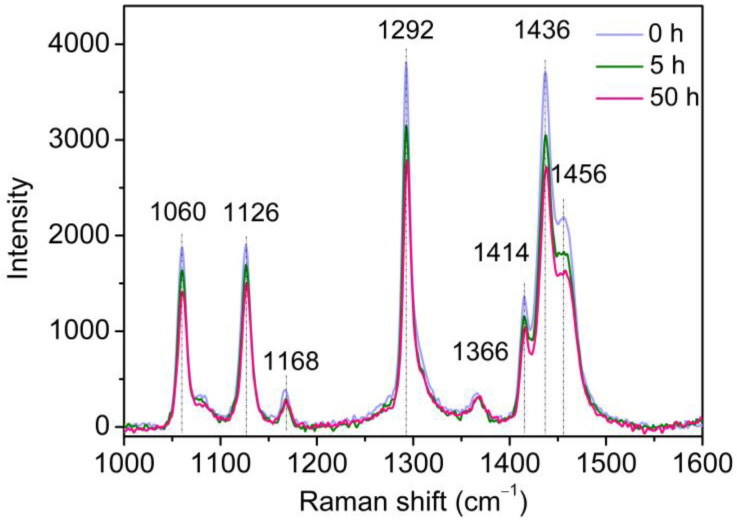
Raman spectra of untreated and corona discharge-treated PE for different duration.

Raman spectra of untreated and corona discharge-treated PE with varying duration are shown in Figure 2. Raman shifts appearing at 1060 cm^−1^, 1126 cm^−1^, 1168 cm^−1^, 1292 cm^−1^, 1336 cm^−1^, 1414 cm^−1^, 1436 cm^−1^ and 1456 cm^−1^ are the characteristic peaks of methylene. The Raman shifts lying at 1060 cm^−1^ and 1126 cm^−1^ are attributed to asymmetric (ν_as_) and symmetric (ν_s_) stretching vibration of C-C bonds, respectively. And the Raman shifts located at 1168 cm^−1^ and 1292 cm^−1^ are attributed to the rocking vibration (ρ) and distortion vibration (τ) of methylene, respectively. The Raman shifts that appear at 1366 cm^−1^ and 1414 cm^−1^ are attributed to the wagging vibration (ω) of methylene. The Raman shifts that appear at 1436 cm^−1^ and 1456 cm^−1^ are attributed to the bending vibration (δ) of methylene. With increasing corona discharge duration from 5 h to 50 h at 5 kV, the characteristic Raman band position is unchanged, but the band intensity decreases obviously. The Raman spectra variations in intensity are in accordance with the ATR-FTIR description, which indicates the successful chemical modification of corona discharge treatment. 

### 2.2. Surface Hydrophilicity of Corona Discharge-Treated PE

**Figure 3:**
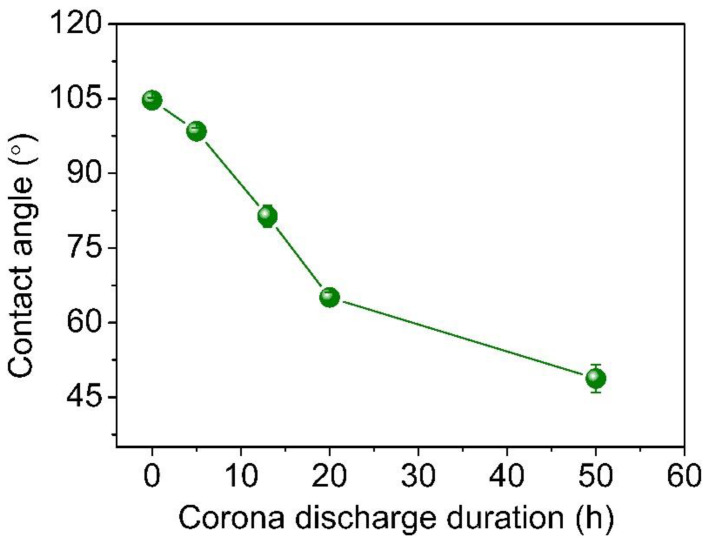
Water contact angle of untreated PE and corona discharge-treated PE.

Figure 3 depicts the water contact angles of untreated and corona discharge-treated PE samples. For the untreated PE and 5-hour-treated sample, the water contact angles are 104.7° and 98.5°, respectively, which exhibits hydrophobicity. With increasing treatment duration from 5 h to 50 h, the water contact angle decreases to 48.8°. After corona discharge treatment, the decrease in the water contact angle indicates a remarkable increase in hydrophilicity, which is explained by the introduction of oxygen-containing polar groups. In order to meet the requirements of dyeability improvement, Park et al. increased the corona discharge power to introduce a polar group and to induce the graft polymerization of acrylic acid onto the corona-treated low-density PE film surface [25]. The results substantiate the enhanced hydrophilicity caused by corona discharge treatment for the variation in chemical character of olefin chain.

### 2.3. Free Volumes Analysis of Corona Discharge-Treated PE

**Figure 4:**
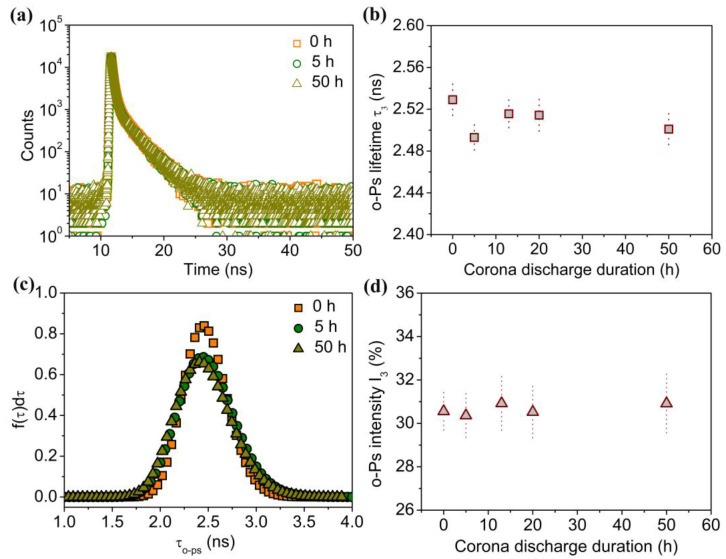
Positron annihilation lifetime spectroscopy analysis of untreated PE and corona discharge-treated PE: (**a**) the positron annihilation lifetime spectra; (**b**) o-Ps lifetime τ_3_ against corona discharge duration; (**c**) distribution of o-Ps lifetime τ_3_; (**d**) o-Ps intensity I_3_ against corona discharge duration.

Figure 4a shows the PALS analysis for untreated and corona discharge-treated PE. After the source component was subtracted and divided into three lifetime components by an LT program, the third lifetime component was attributed to the *o*-Ps. The τ_3_ and I_3_ are closely related to free volumes’ mean size and the relative quantity, respectively. Figure 4b shows the variation in τ_3_ versus corona duration. It is clearly observed that the τ_3_ of untreated PE is ~2.529 ns, and the corresponding mean free volume radius calculated from the Tao–Eldrup equation is 0.329 nm. After corona discharge treatment, τ_3_ shows a slightly decrease, and it is maintained at ~2.510 ns. For conventional PALS, the detection depth is approximately in the tens of microns. The results exhibit average defect information of the corona aging surface and the virgin matrix inside, which could not reflect the information of corona aging region on the surface accurately. As shown in Figure 4c, for untreated PE, the probability density distribution of τ_3_ exhibits a narrow distribution. However, for 5-hour- and 50-hour-treated samples, the probability density distributions of τ_3_ widen and decrease clearly. It can be explained that corona discharge treatment destroys the olefin chain structure and changes the stacking structure. This effect contributes to the broadening of distribution and the decrease in free volume size. Figure 4d shows no significant differences in the *o*-Ps intensity (I_3_) as a consequence of corona discharge treatment duration. For untreated PE, I_3_ is 30.554%. After corona treatment, I_3_ keeps at ~30.5%. Due to the energetic positrons, the implantation depth spans the corona aging surface and the virgin matrix inside. Both the surface and the matrix contribute to the observed positron annihilation information. Therefore, it is necessary to investigate the corona discharge-treated surface and the virgin matrix separately. Another effective detection method should be used to study the depth profile of the microstructure within the corona discharge aging area.

### 2.4. Microstructure Depth Profile of Corona Discharge-Treated PE

**Figure 5:**
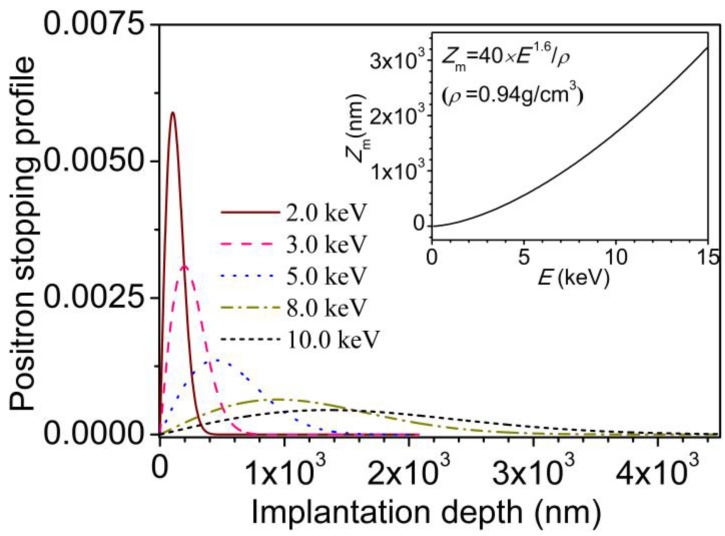
The positron stopping profile in polyethylene with different positron incident energies. The inset shows the relation between positron implantation energy (*E*) and the mean implantation depth (*Z*_m_) (*ρ* = 0.94 g cm^−3^).

The positron stopping profile for a variable energy positron beam (E) equaling 2.0, 3.0, 5.0, and 8.0 keV is calculated and illustrated in Figure 5. The inset shows the mean implantation depth (*Z*_m_) as a function of *E*. As explained elsewhere [26], considering the positron diffusion length in PE within a few nanometers, the stopping profile can be used as the deposition depth profile of positrons. After corona discharge treatment, the depth profile of microstructure can be extensively studied by a slow positron beam with variable energy. The line shape *S* parameter is closely related to the character of overall free volumes or holes. Therefore, the microstructure evolution across the corona discharge damage depth can be extensively studied via the variation in *S* parameter.

**Figure 6:**
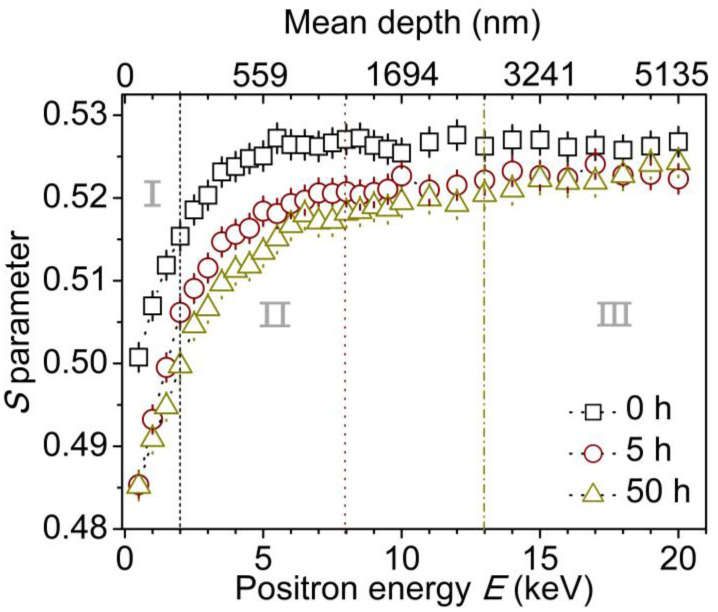
*S* parameters of untreated and corona discharge-treated PE versus the incident positron energy *E* and the mean depth from the surface. The *S-E* curves have been divided into two or three regions (I, II and III) to separate the near surface layer, coronaaging layer and the PE virgin bulk.

Figure 6 shows the depth profile of *S* parameters for different corona discharge-treated PE samples. As a typical *S-E* curve, the *S* parameter exhibits a small value and increases dramatically with increasing positron energy from 0 keV to 2 keV in region I. It is ascribed to the positron annihilation near the surface because of the backing scatting and backing diffusion of the low energy positron or Ps injected close to the surface, which is an intrinsic feature of the slow positron technique [27]. The thickness of the near surface is around 129 nm. And then the *S* parameter increases gradually and keeps at a constant of 0.526 until the positron energy increases to 6.0 keV at a depth of 748 nm (region II). This is because most positrons are implanted in PE bulk and annihilate therein.

After corona discharge treatment, the *S-E* curves possess different characters, and the magnitude of the *S* parameter systematically decreases with increasing corona duration from 5 h to 50 h when positron energy ranges from 0.5 to 20.0 keV. And the *S* parameter reaches a lower plateau after reaching a deeper depth approaching the PE bulk with increasing corona duration. For the 5-hour-treated sample, in region I, similar to the untreated PE and other polymers [28], the *S* parameter exhibits a low value near the surface. In region II, with positron energy increasing up to 8.0 keV, the *S* parameter increases up to a plateau of ~0.520, indicating that much more positrons penetrate into the corona aging layer at a depth of ~1185 nm. In region III, positrons with energy beyond 8.0 keV injected into PE bulk and the *S* parameter keeps at ~0.522, which approaches the value of untreated PE.

For the 50-hour-treated sample, the *S-E* curve presents similar features but also shows a corona-aging region (II) with a thickness of 2577 nm when the positron energy ranges from 2.5 keV to 13.0 keV. Additionally, compared with the other two samples, the lowest *S* parameter indicates that there is a densest structure with smallest free volume size and lowest free volume concentration. The reduction in *S* parameter can be explained as follows: (1) the chain breakage leads to a decrease in free volume size and quantity, so as to a decrease in trapped positronium formation, and (2) the oxygen-containing groups including hydroxyl, carbonyl and ester groups contribute to a decrease in the *S* parameter [27,28,29,30]. Considering that the variation in *S* parameter is directly related to the free volume changes in size or concentration, a lower value of *S* parameter means the size or the concentration of free volumes is lower. It suggests that a dense surface structure with a certain thickness different from the PE virgin bulk is formed after corona discharge treatment. With increasing the corona treatment duration from 5 h to 50 h, the *S* parameter decreases, while the thickness of corona aging layer increases from 1185 nm to 2577 nm. During the corona discharge process, the polyethylene chain structure linked by methylene is broken, and the shorter chains possess a more compact structure than that in the untreated PE. Therefore, the resultant lower free volume size and free volume quantity as well as oxygen-containing polar groups contribute to decreases in the *S* parameter. This is consistent with the results of ATR-FTIR and Raman spectroscopy.

To extensively elucidate the structural changes after corona discharge treatment, the reduction in the *S* parameter, −Δ*S*, is presented and calculated as follows:

(3) $$-\Delta S=-(S_{t}-S_{0})$$

where *S_0_* and *S_t_* are the *S* parameters before and after the specified corona duration, respectively. 

**Figure 7:**
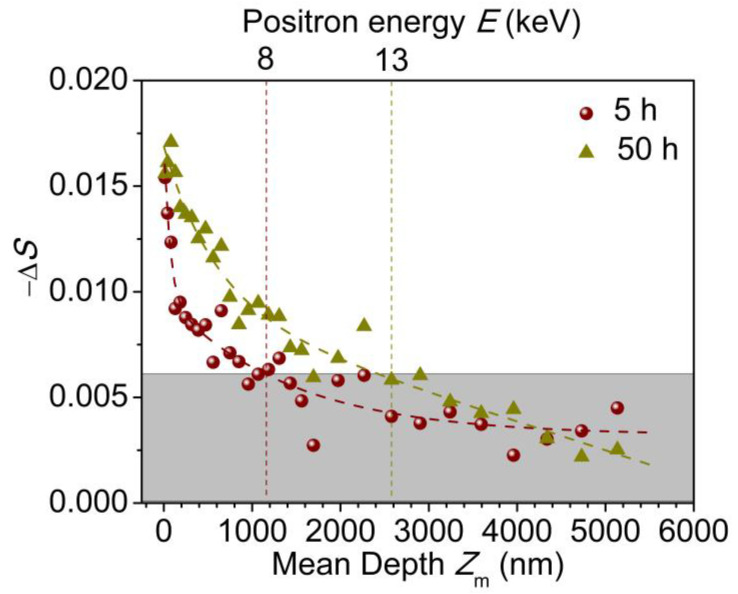
*−ΔS* versus the incident mean depth for 5-hour and 50-hour-treated samples.

Figure 7 shows the −Δ*S* of 5-hour and 50-hour-treated samples versus incident mean depth of monoenergetic positrons beam. And the dashed line is well fitted using a two-exponential decay function. It is clearly observed that the −Δ*S* is larger near the sample surface within 129 nm, which corresponds to the positron injected energy of 2.0 keV. And it decreases gradually with the depth approaching to the PE bulk. In addition, for the 5-hour-treated sample, it drops to a plateau at the position of ~1.2 μm from the surface. Increasing the corona discharge treatment duration from 5 h to 50 h, the *−ΔS* increases, and it keeps consistent with each other beneath the depth of ~2.5 μm from the surface. The profile of *−ΔS* reflects the gradient attenuation of corona discharge treatment with depth. And the degradation moves deeper with increasing corona discharge treatment duration.

**Figure 8:**
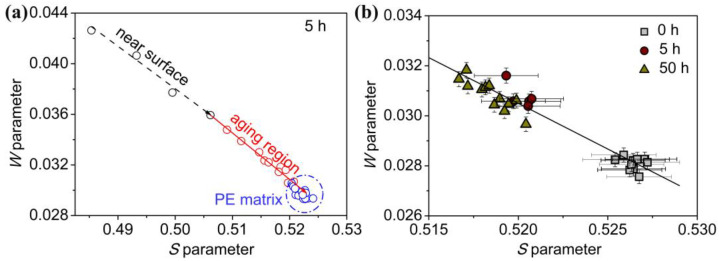
(**a**) *S-W* plot of PE corona discharge treated for 5 h. Arrows show the increasing incident positron energy *E* from 0.5 keV to 20 keV. (**b**) *S-W* plot of PE matrix and corona-aging region.

Generally speaking, the *S-W* plot indicates the interaction between positions and valence electrons, and it can be used to investigate the chemical environments of the positron annihilation trapping sites. Figure 8a shows the linear plot for the specified (*S*, *W*) parameters of a 5-hour-treated sample in different trapping sites along the depth profile. With increasing the incident positron energy, the (*S*, *W*) data basically fall on a straight line. It indicates that the defect type where the positrons are annihilated does not change. The (*S*, *W*) data of (0.512, 0.0339) and (0.522, 0.0296) are attributable to the annihilation of positrons in the corona-aging region and the PE bulk, respectively. Figure 8b depicts the relation among (*S*, *W*) data derived from the annihilation of positrons in the corona-aging region with different corona durations. The *S* parameter decreases and the *W* parameter increases gradually with increases in the corona discharge duration. The lowest *S* value is observed in the 50-hour-treated sample, and the highest value is found in the untreated PE sample. However, these (*S*, *W*) data from different corona durations fall on a straight line. This indicates that the tapping site is same where positrons annihilated but has a different chemical environment. With increasing the corona duration from 5 h to 50 h, changes in the microstructure and chemical environment within the PE depth file result in a decrease in the S parameter and an increase in the W value. On one hand, the hydrocarbon chain scission contributes to the formation of small free volumes and low mobility. Due to the intermolecular crosslinking and shorter bond lengths, the *S* parameter decreases [16]. On the other hand, with increases in the corona duration, the oxygen-containing groups, including hydroxyl, carbonyl and ester groups, are introduced. Compared with the hydrocarbon chain, positron annihilation in the proximity of an oxygen environment contributes to the trapping of positrons. Positron annihilation with high-momentum core electrons belonging to oxygen is enhanced. A lower S parameter is obtained [27]. Therefore, positron is an effective probe to detect the depth profile of microstructures and chemical characteristics in the surface and interfacial study.

## 3. Materials and Methods

**Figure 9:**
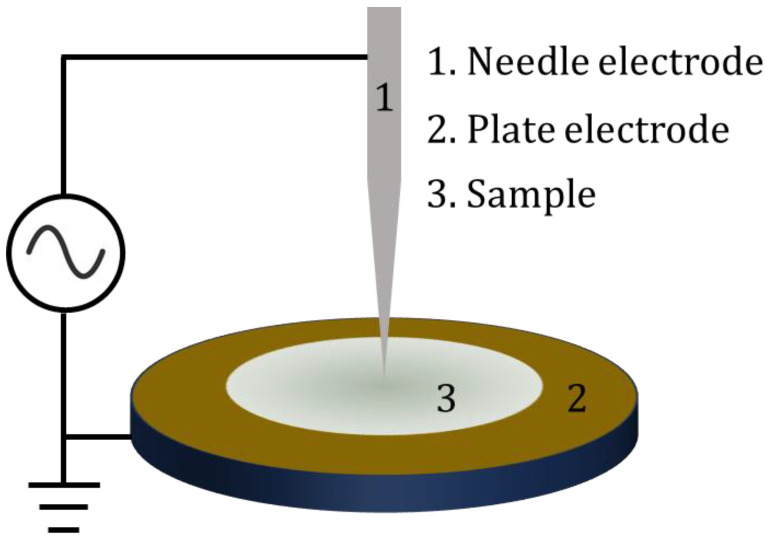
The “needle-plate” electrodes system.

Preparations: PE plate samples were prepared using a hot-pressing method with dimensions of 30 mm × 30 mm × 10 mm. And the density was 0.94 g·cm^−3^. As illustrated in Figure 9, the corona discharge setup consisted of a “needle-plate” electrode system, as described in our previous studies [31,32]. Both the needle and plate electrodes were made of 304 stainless steels. The diameter of the needle electrode was 3 mm, with a curvature radius of the needle tip measuring 50±3 um. The cylinder plate electrode had a diameter of 20 mm and a thickness of 5 mm. During the corona discharge treatment, the experiments were conducted in air at atmospheric pressure and room temperature, with humidity below 50%. An alternating voltage of 5 kV at 50 Hz was applied to the needle electrode while the plate electrode was grounded. Samples were positioned at the center of the plate electrode with a distance of 2 mm between the specimen surface and the needle. The durations for corona discharge treatment were 5 h, 13 h, 20 h and 50 h, respectively.

Characterizations: The chemical characteristics of untreated and corona discharge-treated PE samples were investigated using ATR-FTIR and Raman spectroscopy. The ATR-FTIR spectra were acquired using an FTIR spectrophotometer (Nicolet 170 SXIR; Waltham, MA, USA). Raman spectra were acquired using a Laser Confocal Raman Microspectroscopy (LabRAM HR800, Waltham, MA, USA) with a laser wavelength of 488 nm over the range of 4000 cm^−1^–500 cm^−1^. The spot diameter was 1~2 μm, and the CCD exposure time was 10 s. Water contact angle (WCA) measurements were utilized to characterize the hydrophilicity variations. The water contact angle of each sample was measured by a sessile drop method using a goniometer (SL200 KB Kino, Boston, MA, USA) with an accuracy of ±3θ. And the volume of each droplet of deionized water was 3 μL. Five locations were examined on each sample. All the PALS measurements in this work were conducted under ambient conditions and were carried out utilizing a conventional fast–fast coincidence system with a time resolution of 280 ps (full width at half-maximum). During the measurements, two identical samples with a thickness of ~1 mm were sandwiched together with a 20 μCi positron source (^22^NaCl) sealed with 7 μm thick Kapton films, and each lifetime spectrum was accumulated with a total of one million counts. All of the PALS spectra were evaluated using the LT routine [33] after source correction using Ni signal crystal as a reference. All the spectra were decomposed into three lifetime components without fixing any lifetime or intensity, and the variances of the fits were in the range of 0.95~1.10. The depth profile of microstructure was investigated by DBS with a high-purity germanium detector with a monoenergetic positron beam ranging from 0.5 keV to 20 keV at the Institute of High Energy Physics Chinese Academy of Science, Beijing, China. A spectrum with 5 × 10^5^ counts was collected for each incident positron energy. The line shape parameter *S* for 511 keV γ-annihilation peak is defined as the ratio of the annihilation events of the central region (511 keV ± 0.75 keV) to the total area of the peak (499.5–522.5 keV). The line shape parameter *W* is defined as the ratio of the annihilation events of the wing regions (505.10–508.40) keV and (513.6–516.9) keV to the total area of the peak.

## 4. Conclusions

In conclusion, the depth profile of microstructures and chemical properties of the corona discharge-treated polyethylene were investigated using positron annihilation spectroscopy accompanied with ATR-FTIR, Raman spectroscopy and water contact angle. By increasing corona discharge duration, the formation of the oxygen-containing polar groups including hydroxyl, carbonyl, and ester groups, together with the breakage of the hydrocarbon chain, strongly contribute to the deterioration of hydrophobicity and the enhancement of hydrophilicity. In addition, the mean free volume size, with a broadening of the probability density distribution, decreases with increases in the corona duration. Also, the thickness of the degradation layer, determined from the *S* parameter with positron injection depth, increases and diffuses into the PE matrix. Increasing the corona discharge duration from 5 h to 50 h, the thickness of corona degradation layer ranges from ~1.2 μm to ~2.5 μm, which is proved by the *S-E* curves and the fitting results from the *−ΔS*-*Z*_m_ curves. At the same time, a linear *S-W* plot within the degradation layer of different corona discharge-treated samples indicates the defect type does not change. The *S* parameter decreases and the *W* parameter increases with an increasing corona duration because of the hydrocarbon chain’s scission and the oxygen-containing groups. The chemical variation during the corona discharge process can be clearly identify by positron annihilation. Using a slow positron beam, the nondestructive probe can be used to profile the microstructure and chemical environment across the corona discharge damage depth, which is beneficial to investigate the surface and interfacial insulation materials a function of depth from the surface.

## Data Availability

Data are contained within the article.
